# CRISPR knock out of programmed cell death protein 1 enhances anti-tumor activity of cytotoxic T lymphocytes

**DOI:** 10.18632/oncotarget.23730

**Published:** 2017-12-27

**Authors:** Zhilong Zhao, Long Shi, Wei Zhang, Jinsheng Han, Shaohui Zhang, Zexian Fu, Jianhui Cai

**Affiliations:** ^1^ Department of Surgery, Hebei Medical University, Shijiazhuang, Hebei, China; ^2^ Department of Oncology, Hebei Medical University Second Affiliated Hospital, Shijiazhuang, Hebei, China; ^3^ Department of Surgery & Oncology, Hebei General Hospital, Shijiazhuang, Hebei, China; ^4^ Department of Surgery, Handan Central Hospital, Handan, Hebei, China; ^5^ Department of Surgery, Cangzhou Hospital of Traditional Chinese Medicine and Western Medicine Integrated Hebei, Cangzhou, Hebei, China; ^6^ Department of Oncology, Hebei Medical University Third Affiliated Hospital, Shijiazhuang, Hebei, China; ^7^ Department of Oncology, Hebei University of Engineering Affiliated Hospital, Baoding, Hebei, China

**Keywords:** anti-tumor immunity, CRISPR, cytotoxic T lymphocyte, immune checkpoint, PD-1

## Abstract

Programmed cell death protein 1 (PD-1) is an immune checkpoint receptor that functions to attenuate T cell activation. In this study, we knocked out (KO) PD-1 in cytotoxic T lymphocytes (CTLs) using CRISPR-Cas9 system to evaluate its effect on the anti-tumor activity of the CTLs against multiple myeloma (MM). Results show that PD-1 KO CTLs facilitate apoptosis and caspase activation of the co-cultured MM cells and enhanced MM cell death by 36% compared with the control. PD-1 KO also increased TNF-α and IFN-γ secretion of the CTLs by 2.4 and 1.9-fold respectively. The effectiveness of PD-1 KO in enhancing anti-tumor activity of the CTLs was verified *in vivo* using mouse xenograft model. The xenografted mice treated with PD-1 KO CTLs demonstrated repressed MM tumor growth and prolonged survival compared with the control group. We conclude that CRISPR-Cas9 is an efficient system to knock out PD-1 from CTLs and PD-1 KO could significantly enhance the anti-tumor activity of CTLs.

## INTRODUCTION

The immune system plays a critical role in cancer development. Under normal circumstances, the immune system can recognize, control, and even eliminate tumors [1]. Cytotoxic T lymphocytes (CTLs, also known as CD8+ T cells or killer T cells) are the primary immune cells responsible for killing tumor cells. T cells express programmed cell death protein 1 (PD-1) on their surface [2]. PD-1, when bound to its ligand PD-L1, generates inhibitory signals to repress T cell activity [3]. PD-L1 is expressed by most types of cells [4]; the PD-1/PD-L1 pathway plays a crucial role in the immune system to keep the self-tolerance and physiological immune responses in balance [5]. Many tumor cells also express PD-L1; tumor cells can utilize the PD-1/PD-L1 pathway to attenuate T cell activity and thus escape the recognizing and killing capacity of CTLs [5]. In this situation, the PD-1/PD-L1 checkpoint functions to interrupt cancer immunity cycle [6].

Regulating effector T cell activity in response to tumor cells is one of the main functions of PD-1 [7]. Increased levels of PD-1 have been reported to be associated with exhausted or chronically stimulated T cells and poor survival in patients [8]. PD-1/PD-L1 pathway blockade is an effective therapy for cancers and has gained remarkable progress in treating patients with lung cancer and advanced hematologic malignancies [7, 9–11]. In this study, we disrupted PD-1/PD-L1 pathway thought editing out PD-1 (PD-1 KO) in CTLs with Clustered regularly interspaced short palindromic repeats (CRISPR) and CRISPR-associated protein 9 (Cas9) system. We then investigated the effect of PD-1 KO on the anti-tumor activity of T cells. Our data shows that the CRISPR-Cas9 system could efficiently knock out PD-1 in CTLs and the knockout of PD-1 enhanced the cytotoxic effect on tumor cells by the CTLs. Our study provides the foundation for the potential application of CRISPR technology in immune checkpoints targeting.

## RESULTS

### Isolation of cytotoxic T lymphocytes (CTLs) and CRISPR knockout (KO) PD-1

**Figure 1:**
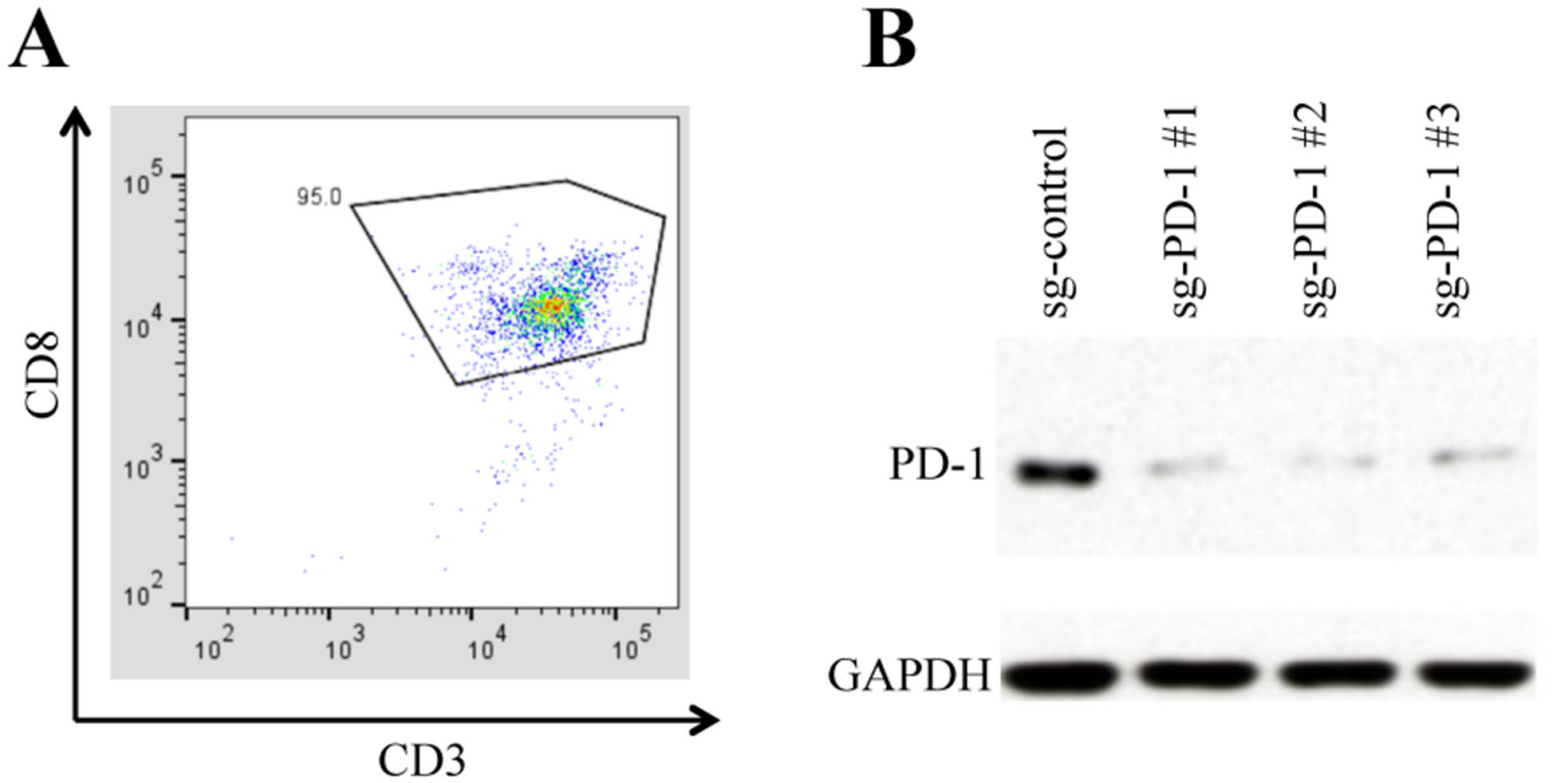
CTLs isolation and PD-1 knockout **(A)** The percentage of CD3+/CD8+ cells (CTLs) reached 95% after negative selection. **(B)** The CTLs were transduced with lentivirus co-expressing Cas9 and each of the indicated guide RNA. The expression of PD-1 guide RNA reduced the PD-1 expression in the transduced cells.

The immunophenotypes of the CTLs isolated from unattached PBMCs were analyzed by flow cytometry to verify the efficiency of cell isolation. The purity of CD3+/CD8+ cells (CTLs) reached 95% (Figure 1A), which is high by negative selection. The PD-1 expression in the lentivirus transduced CTLs was assayed by western blot. The CRISPR-Cas9 system significantly reduced the overall PD-1 expression in the presence of PD-1 guide RNAs (Figure 1B), indicating the efficient PD-1 KO in the transduced CTLs.

### Enhanced anti-tumor cytotoxicity by the disruption of PD-1 in primary CTLs

**Figure 2:**
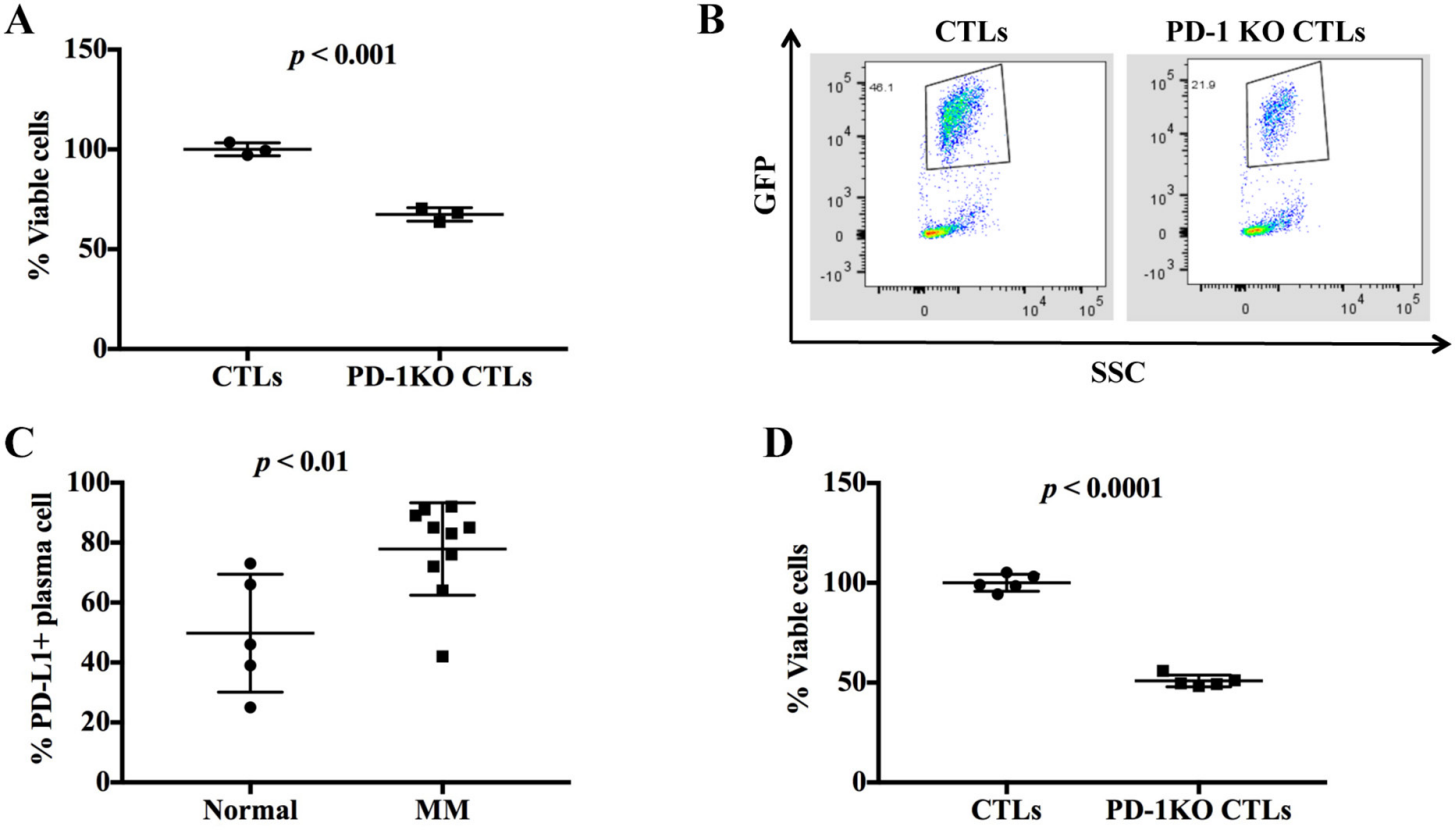
PD-1 disruption increased the cytotoxic activity of CTLs **(A)** The cell viability of MM.1S co-cultured with the control or PD-1 KO CTLs were compared and normalized to the control. **(B)** The MM cell survival was assessed by the percentage of GFP positive cells. **(C)** Multiple myeloma (MM) patients have more PD-L1 expressing plasma cells than the normal healthy donors. **(D)** The viability of purified patient CD138+ cells (MM cells) in the control and PD-1 KO co-culture was compared and normalized to the control.

We next tested if the disruption of PD-1 could enhance the anti-tumor activity of the CTLs against multiple myeloma (MM). We first assayed the cytotoxicity of PD-1 KO CTLs on MM.1S cancer cell line. After being co-cultured with CTLs (control or PD-1 KO) for 24 h, the MM.1S cells were measured for cell viability. WST-1 results showed that the MM.1S cell viability in PD-1 KO co-culture decreased to 63.49 ± 2.71% of that in the control co-culture (Figure 2A). Flow cytometry analysis also demonstrated a reduction of tumor cells in the PD-1 KO co-culture: the MM.1S cells accounted for 46.1% in the control co-culture but only 21.9% in the PD-1 KO co-culture (Figure 2B). MM patients have accumulated malignant plasma cells (CD138+ cells) in the bone marrow and the increased PD-L1 expression in plasma cell has been related to MM development [12]. In our study, the MM patients had more PD-L1 expressing plasma cells (77.53 ± 8.27%) than normal healthy donors (49.69 ± 19.01%) (Figure 2C). To determine the effect of PD-1 KO CTLs on patient MM cells, we incubated patient CD138+ cells (MM cells) with CTLs. Similar as in MM.1S cell line, PD-1 KO CTLs caused about 50% more patient MM cell death than did the control CTLs (Figure 2D). These data clearly show that PD-1 KO enhanced the cytotoxicity of CTLs.

### PD-1 KO in CTLs enhanced apoptotic and caspase activities in tumor cells

**Figure 3:**
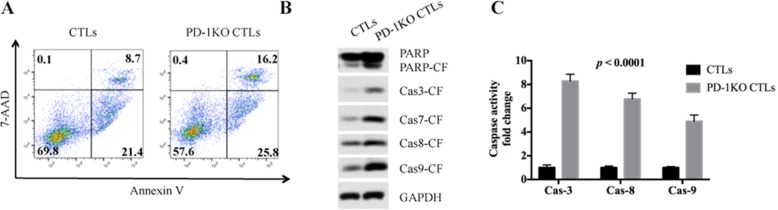
PD-1 disruption in CTLs increased MM cell apoptosis and caspase activities **(A)** Annexin V/7-AAD staining shows that MM.1S cells co-cultured with PD-1 KO CTLs have higher level of early stage and late stage apoptotic cells. Annexin V+/7-AAD- means early apoptotic, Annexin V+/7-AAD+ means late apoptotic. **(B)** Compared to the control, the MM.1S cells co-cultured with PD-1 KO CTLs demonstrated higher level of the cleaved form (CF) of PARP, caspase-3, caspase-7, caspase-8 and caspase-9. **(C)** Compared to the control, the MM.1S cells co-cultured with PD-1 KO CTLs demonstrated higher level of caspase enzymatic activity.

The apoptosis of MM.1S cells were analyzed by Annexin V/7-AAD staining followed by flow cytometry analysis after being co-cultured with CTLs for 12 h. 16.2% of MM.1S cells entered late apoptotic stage in the PD-1 KO co-culture, whereas only 8.7% of MM.1S cells entered this stage in the control co-culture (Figure 3A). Accordingly, the percentage of early apoptotic MM.1S cells in the PD-1 KO and control co-culture are 25.8% and 21.4% respectively. These data clearly show that PD-1 KO CTLs induced a higher level of apoptosis in the co-cultured tumor cells than did the control CTLs.

Caspases play essential roles in programmed cell death. Caspases are synthesized as inactive procaspase and are activated by proteolytic cleavage during cell apoptosis [13]. We found the MM.1S cells in the PD-1 KO co-culture have more cleaved form of caspase-3, caspase-7, caspase-8, caspase-9 and PARP than the control (Figure 3B), showing that PD-1 KO CTLs lead to an elevated caspase activation in tumor cells. We then incubated MM.1S cell lysates with different caspase substrates to test their caspase activities. Results confirmed that the MM.1S cells’ caspase-3, caspase-8, and caspase-9 activities in PD-1 KO co-culture are respectively 8.21±0.47, 6.58±0.41 and 4.92±0.35 times higher than that of the control (Figure 3C).

### Increased cytokine secretion by the disruption of PD-1 in primary T cells

**Figure 4:**
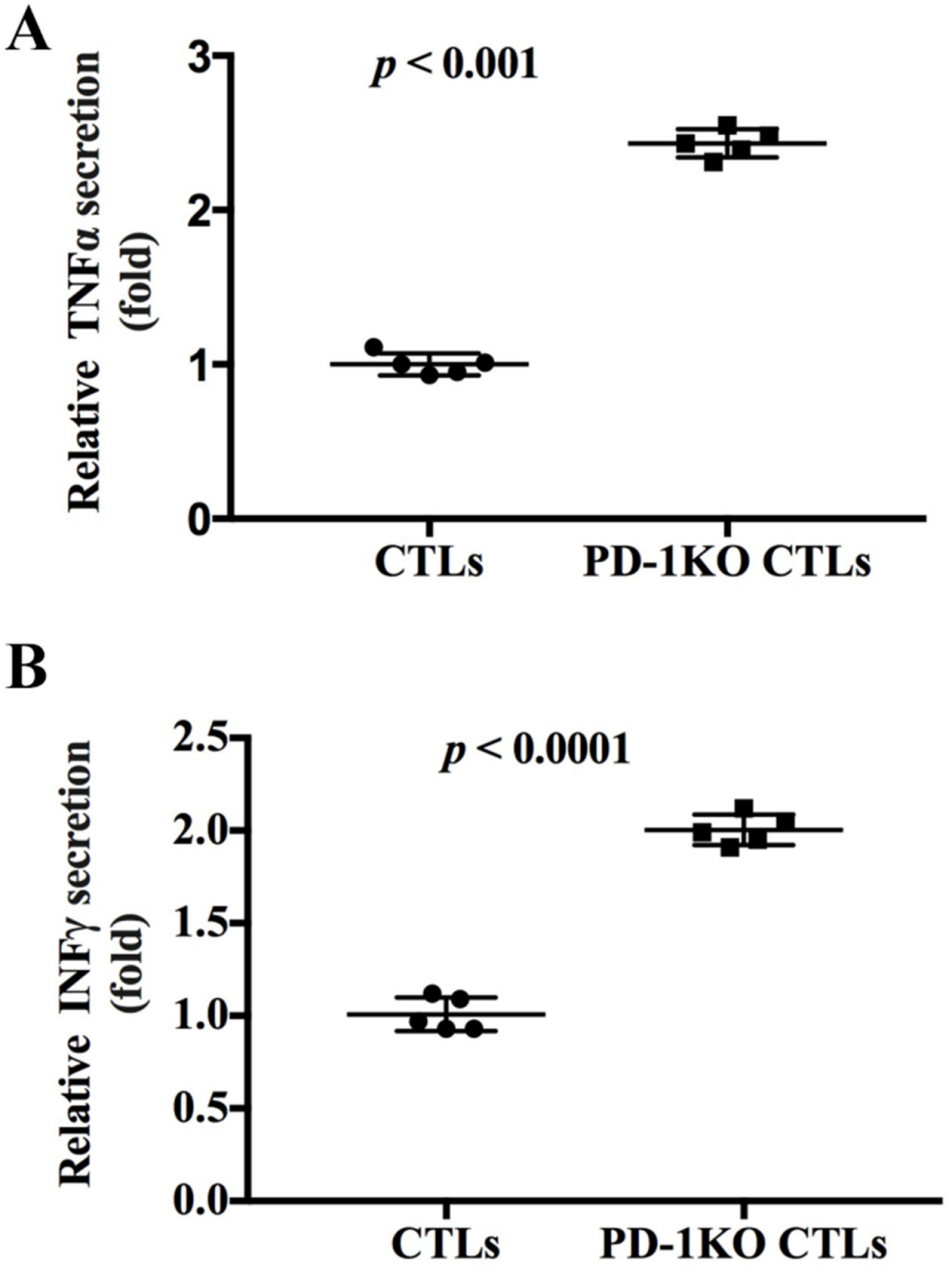
PD-1 knockout increased the secretion of TNF-α and IFN-γ by CTLs **(A)** The secretion of TNF-α in the control and PD-1 KO CTLs were tested and normalized to control CTLs. **(B)** The secretion of IFN-γ in the control and PD-1 KO CTLs were tested and normalized to control CTLs.

CTLs produce cytokines as an effector mechanism to kill viral infected cells and tumor cells. The cytokines produced by CTLs include TNF-α and IFN-γ. Since we found that PD-1 KO enhanced the anti-tumor activity of the CTLs, we next explored if PD-1 KO could enhance the cytokines secretion of CTLs. ELISA assays showed that PD-1 KO CTLs secreted 2.43±0.18 and 1.92±0.21 times more TNF-α and IFN-γ respectively than did the control CTLs (Figure 4A and 4B), showing that PD-1 KO could increase the cytokines production of CTLs.

### PD-1 knockout CTLs inhibit MM cell growth in human xenograft mouse model

**Figure 5:**
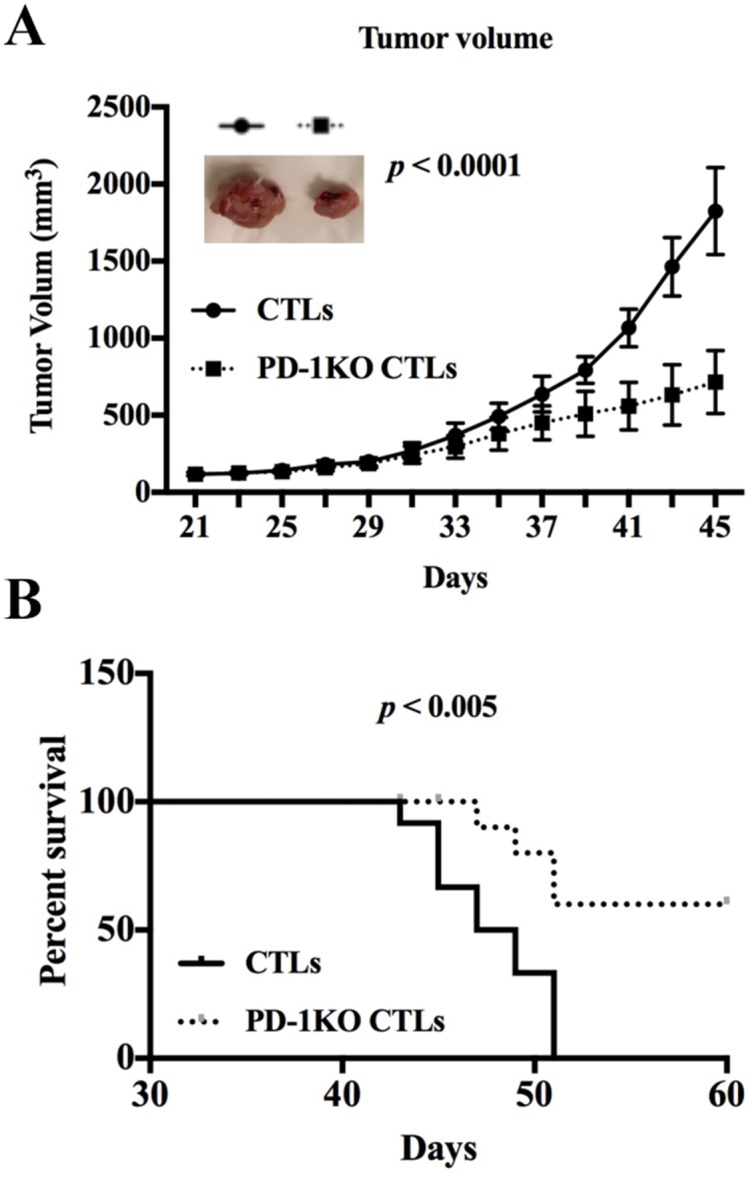
PD1 KO CTLs repress tumor growth more efficiently than the control CTLs in xenografted mice **(A)** The tumor growth in human MM.1S bearing mice treated with either control or PD-1 KO CTLs. A representative tumor picture shows the repressed tumor growth in the mouse treated with PD-1 KO CTLs. **(B)** Kaplan–Meier plot shows survival in mice treated with either control or PD-1 KO CTLs.

The efficacy of PD-1 KO CTLs in repressing tumor growth was validated *in vivo* using human tumor xenograft model. Two groups of CB-17 SCID mice were subcutaneously inoculated with 5 × 10^6^ MM.1S cells following 200 rad irradiation. The mice were then treated with either control CTLs (control group) or PD-1 KO CTLs four different times to evaluate the repression of CTLs on tumor growth. The tumor became detectable after 2.5 weeks and the tumor growth in the PD-1 KO CTLs treated mice was dramatically repressed compared to that in the control group (Figure 5A). All the mice in the control group died from progressive tumors by 52 days. In contrast, only 40% of PD-1 KO CTLs treated mice died at the same time (Figure 5B). Thus, PD-1 KO in CTLs effectively inhibited human MM cell growth *in vivo* and significantly improved overall survival of the xenografted mice.

## DISCUSSION

Engagement of PD-1/PD-L1 pathway leads to inhibition of T-cell effector function. It has already been reported that blocking PD-1/PD-L1 pathway by monoclonal antibodies increased T cell-mediated cytotoxicity [14–16]. In this study, we disrupted PD-1 expression in cytotoxic T lymphocytes (CTLs) using CRISPR-Cas9 system and tested the anti-tumor effect of PD-1 knockout (KO) CTLs on multiple myeloma cells. Compared to the control CTLs, PD-1 KO CTLs were found to be more efficient at killing tumor cells. The anti-tumor activity of PD-1 KO CTLs was verified *in vivo* using mouse xenograft model. The treatment with PD-1 KO CTLs significantly inhibited xenograft tumor growth and prolonged the overall survival of the host. The mechanistic study showed that the enhanced anti-tumor effect of PD-1 KO CTLs is associated with increased apoptosis and augmented caspase activities in tumor cells. The secretion of cytokines (primarily TNF-α and IFN-γ) is one of the mechanisms by which CTLs kill tumor cells. PD-1 KO increased the cytokines secretion by CTLs, which could be accounted in part by the increased anti-tumor activity of PD-1 KO CTLs. This study expands the arsenal of cancer therapy by showing the potential application of CRISPR technology in targeting PD-1 immune checkpoint.

## MATERIALS AND METHODS

### Cell culture and reagents

Human multiple myeloma (MM) cell line MM.1S and peripheral blood mononuclear cells (PBMCs) from normal healthy donors and patients were cultured in RPMI-1640 medium supplemented with 10% FBS and antibiotics.

### Dendritic cell (DC) preparation

Peripheral blood samples were obtained from healthy donors from Hebei Blood Center, Hebei, China. Informed consent was obtained from all donors in accordance with the guidelines verified and approved by Hebei Medical University, Hebei, China. PBMCs were isolated from whole blood samples by Ficoll-Paque density gradient centrifugation (GE Healthcare, Chicago, IL, USA). Mononuclear cells from the interphase were collected, washed with complete medium three times and then cultured at 37°C with 5% CO_2_. After two hours, unattached cells and medium were collected for cytotoxic T lymphocytes (CTLs) isolation. Attached cells were washed and then cultured in RPMI complete medium supplemented with 100 ng/ml human recombinant Granulocyte-macrophage colony-stimulating factor (GM-CSF, PeproTech, America) and 100 ng/ml human recombinant IL-4 (PeproTech, America) for 6 days. On day 5, irradiated MM tumor lysates were added into cultured DCs. After 24h, 25 ng/ml IL-1β and 100 ng/ml TNF-α were added into the culture for another 24 h. Mature DCs were collected on day 7.

### CTLs isolation and activation

CTLs were isolated from unattached PBMCs using Dynabeads Untouched Human CD8 T cells Kit (Thermo Fisher Scientific, Waltham, MA, USA) following manufacturer's protocol. The isolated CD8+ T cells were activated by pre-loaded DCs at the ratio of DC: T = 1:10. Mixed-culture was incubated in RPMI complete medium supplemented with 50U/ml recombinant human IL-2 for 3-4 days to keep cell expansion.

### CRISPR/Cas9 lentivirus production

The non-targeting guide RNA sequence and three PD-1 guide RNA sequences were designed using online program http://crispr.mit.edu. The PD-1 guide RNA sequence PD-1sg-1: aggcgccctggccagtcgtc, PD-1sg-2: cgtctgggcggtgctacaac, PD-1sg-3: ctacaactgggctggcggcc and the non-targeting control RNA sequence: atcgtttccgcttaacggcg were respectively cloned into LentiCrispr v2 vector at BsmBI site following the protocol described previously [17]. Lentivirus was produced by co-transfecting 293T cells with the packaging vector ps-PAX2, the envelope vector pCL-VSVG and each of the LentiCrispr V2 vectors. 24 h after transfection, the culture medium was changed to RPMI supplemented with 10% FBS. The lentivirus in the supernatant was collected 72 h after transfection and filtered through 0.45 μm syringe filter.

### Generation of PD-1 KO cell lines

CTLs were transduced with lentivirus expressing Cas9 and guide RNA 24 h before DC activation. After co-culture with DCs, RPMI growth medium was changed to selection medium containing 1 μg/ml puromycin for 3 days. Cells were then expanded and frozen down once the cells were growing normally in selection medium.

### Fluorescence-activated cell sorting (FACS) analysis

Flow cytometry was performed on BD Canto with FACS Diva software (BD Biosciences, San Jose, CA, USA). Samples were analyzed with FlowJo v.10.2 (TreeStar, Ashland, OR, USA). The CTLs isolated from unattached PBMCs were incubated with fluorescence-labeled CD3 and CD8 antibodies before being subjected to FACS analysis. Mononuclear cells isolated from bone marrow by centrifugation on a Ficoll density gradient were stained with CD138 and PD-L1 antibodies and subjected to FACS analysis. The MM cells that express green fluorescence proteins (GFP) were FACS-analyzed directly after being co-cultured with CTLs. For the cell apoptosis assay, the apoptotic cells were stained with Annexin V-PE and 7-AAD (Biolegend, San Diego, CA, USA) and analyzed by FACS.

### Immunoblotting

Total cell lysates were subjected to SDS-PAGE followed by immunoblotting with indicated antibodies including PD-1(ab52587, abcam), GAPDH (AB2302, EMD Millipore), Caspase-3 (9662, Cell Signaling Technology: CST), Caspase-7 (9494, CST), Caspase-8 (1C12, CST), Caspase-9 (9502, CST) and PARP (9532, CST). Blots were then developed by enhanced chemiluminescence (Bio-rad, Hercules, CA, USA).

### WST-1 assay

MM.1S cells were seeded in triplicate wells in 96-well plates at the density of 2.5 × 10^4^/well. Control or PD-1-KO CTLs were added to the plates at the density of 2.5 × 10^5^/well. Cells were co-cultured for 24 h at 37°C. MM cell viability was assessed by WST-1 (Takara, Katsushika, Tokyo, Japan) assay following manufacturer's protocol. Briefly, 10 μl of WST-1 reagent was added into each well of the cell culture and incubated at 37°C for 1 h. Absorbance at wavelength 450 nm was read by standard microplate reader, and data were recorded and normalized by T cell culture alone.

### Caspase enzymatic activity assay

Caspase assay buffer and caspase substrates were purchased from Enzo Life Science, Inc. (Farmingdale, NY, USA). Caspase-3 substrate (Ac-DMQD-AMC), caspase-8 substrate(Ac-LETD-AFC) and caspase-9 substrate (Ac-LEHD-AFC) were used to test the activity of caspase-3, caspase-8 and caspase-9 respectively following the manufacture's instruction.

### Cytokine secretion

TNF-α and IFN-γ secretion were measured with Human TNF-α ELISA Kit (Abcam) and IFN-γ Human ELISA Kit (Thermo Fisher Scientific). Cells were cultured for 5h in complete medium with 100 ng/ml phorbol myristate acetate (PMA, Sigma-Aldrich, St. Louis, MO, USA) and 1 μg/ml ionomycin (Sigma-Aldrich) [18]. After centrifuging at 1,000g for 10 min to remove cells, 50 μl of supernatants were collected and used for ELISA assay following the manufacturer's protocol.

### Human xenograft mice model

All the animal experiments were approved by and conformed to the relevant regulatory standards of Hebei Medical University Animal Care and Use Committee Hebei, China. CB-17 SCID mice (5 weeks old) were irradiated (200 rad) before being subcutaneously inoculated with 5 × 10^6^ MM.1S cells in 100 μl of phosphate-buffered saline. Two groups of mice received either 1 × 10^7^ human CTLs or PD-1 KO CTLs through tail vein on day 1, 2, 3, and 6 following tumor inoculation (10 mice per group). Tumor size was measured using a caliper every other day. The mean volume and SD of each group were calculated. The tumor volume was calculated as 1/2ab^2^ (a = length, b = width). Mice were killed when tumor volume reached 2000 mm^3^. The overall survival was analyzed by the Kaplan–Meier method and calculated from the day of tumor cell injection to the day a mouse was found dead or killed. Experiments were independently repeated for 3 times.

### Statistical analysis

Statistical significance was determined using the Student's *t* test. The results were expressed as mean ±standard deviation. The GraphPad Prism software was used for all statistical analysis. Two-sided p values less than 0.05 were considered statistically significant.
